# ncFO: A Comprehensive Resource of Curated and Predicted ncRNAs Associated with Ferroptosis

**DOI:** 10.1016/j.gpb.2022.09.004

**Published:** 2022-09-23

**Authors:** Shunheng Zhou, Yu’e Huang, Jiani Xing, Xu Zhou, Sina Chen, Jiahao Chen, Lihong Wang, Wei Jiang

**Affiliations:** 1 Department of Biomedical Engineering, Nanjing University of Aeronautics and Astronautics, Nanjing 211106, China; 2 Department of Pathophysiology, School of Medicine, Southeast University, Nanjing 210009, China

**Keywords:** Ferroptosis, microRNA, Long non-coding RNA, Circular RNA, Cancer

## Abstract

**Ferroptosis** is a form of regulated cell death driven by the accumulation of lipid hydroperoxides. Regulation of ferroptosis might be beneficial to **cancer** treatment. Non-coding RNAs (ncRNAs) are a class of RNA transcripts that generally cannot encode proteins and have been demonstrated to play critical roles in regulating ferroptosis. Herein, we developed ncFO, the ncRNA–ferroptosis association database, to document the manually curated and predicted ncRNAs that are associated with ferroptosis. Collectively, ncFO contains 90 experimentally verified entries, including 46 **microRNAs** (miRNAs), 21 **long non-coding RNAs** (lncRNAs), and 17 **circular RNAs** (circRNAs). In addition, ncFO also incorporates two online prediction tools based on the regulation and co-expression of ncRNA and ferroptosis genes. Using default parameters, we obtained 3260 predicted entries, including 598 miRNAs and 178 lncRNAs, by regulation, as well as 2,592,661 predicted entries, including 967 miRNAs and 9632 lncRNAs, by ncRNA–ferroptosis gene co-expression in more than 8000 samples across 20 cancer types. The detailed information of each entry includes ncRNA name, disease, species, tissue, target, regulation, publication time, and PubMed identifier. ncFO also provides survival analysis and differential expression analysis for ncRNAs. In summary, ncFO offers a user-friendly platform to search and predict ferroptosis-associated ncRNAs, which might facilitate research on ferroptosis and discover potential targets for cancer treatment. ncFO can be accessed at http://www.jianglab.cn/ncFO/.

## Introduction

Cell death plays a critical role in tissue development and homeostasis and prohibits the hyperproliferation of cancer cells [1]. Ferroptosis is a particular type of regulated cell death induced by lipid peroxidation [2]. Triggering the ferroptosis process might effectively kill cancer cells and might be a powerful strategy for cancer therapy [3], [4], [5]. Thus, exploring the potential regulators of ferroptosis would be helpful for elucidating the molecular mechanism and scrutinizing the potential drug targets.

Non-coding RNAs (ncRNAs) are a class of transcripts that generally have no capability for protein encoding. In addition, ncRNAs can be divided into several classes according to their length and shape, such as microRNAs (miRNAs), long non-coding RNAs (lncRNAs), and circular RNAs (circRNAs) [6]. Moreover, ncRNAs have been verified to play critical regulatory roles in many biological processes and diseases [7], [8], including ferroptosis [9]. Recently, the FerrDb database curated the genes and small molecules involved in ferroptosis [10]. However, an overwhelming number of publications have reported that ncRNAs participate in the regulation of ferroptosis. The scattered information was inconvenient for researchers to characterize the ferroptosis-associated ncRNAs from a comprehensive perspective.

To fill this gap, we developed the ncRNA–ferroptosis association database (ncFO, https://www.jianglab.cn/ncFO/). ncFO collected the experimentally verified ncRNAs that participate in ferroptosis through manual curation. Meanwhile, to inspect the potential ncRNAs involved in ferroptosis, we proposed “Prediction tools” to identify ferroptosis-associated ncRNAs through ncRNA regulation and co-expression analysis. In addition, the ncFO database also provided survival analysis and differential expression analysis for ncRNAs. We believe that the database might provide a reliable resource for browsing and analyzing ferroptosis-associated ncRNAs and underlining potential targets for cancer therapy.

## Data collection and processing

### Experimentally verified data collection

**Figure 1:**
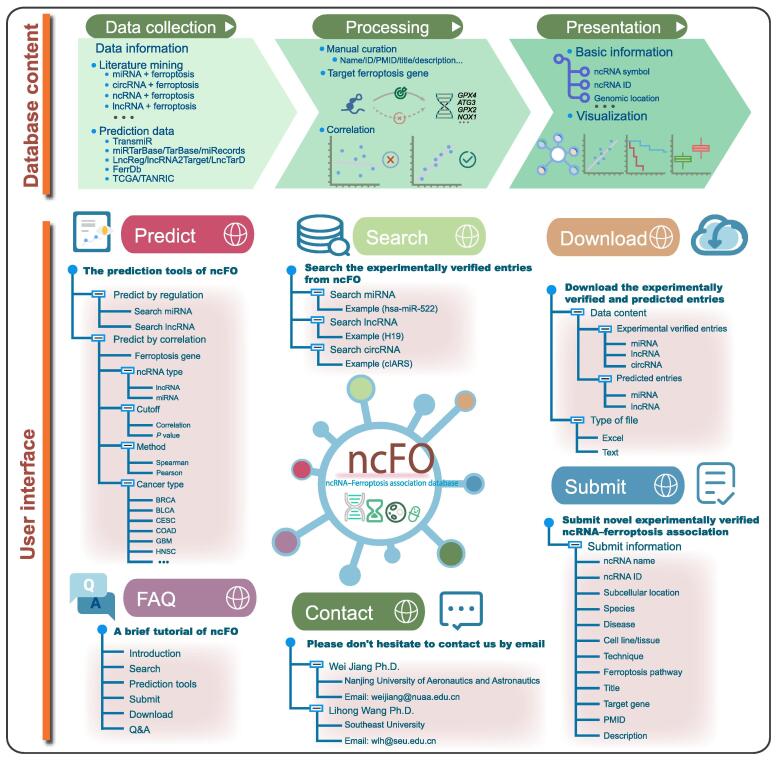
**Workflow for the construction of ncFO database** miRNA, microRNA; circRNA, circular RNA; ncRNA, non-coding RNA; lncRNA, long non-coding RNA; TCGA, The Cancer Genome Atlas; ID, identifier; PMID, PubMed identifier; FAQ, frequently asked question; BRCA, breast invasive carcinoma; BLCA, bladder urothelial carcinoma; CESC, cervical squamous cell carcinoma and endocervical adenocarcinoma; COAD, colon adenocarcinoma; GBM, glioblastoma multiforme; HNSC, head and neck squamous cell carcinoma.

To collect experimentally verified ferroptosis ncRNAs, we queried publications on ncRNAs and ferroptosis from the PubMed database using a list of keywords, including “ferroptosis and miRNA” or “ferroptosis and microRNA” or “ferroptosis and circular RNA” or “ferroptosis and circRNA” or “ferroptosis and lncRNA” or “ferroptosis and long noncoding RNA” or “ferroptosis and long non-coding RNA” or “ferroptosis and lincRNA” or “ferroptosis and long intergenic noncoding RNA” or “ferroptosis and long intergenic non-coding RNA” or “ferroptosis and ncRNA” or “ferroptosis and noncoding RNA” or “ferroptosis and non-coding RNA”. Approximately 200 articles were downloaded from the PubMed database (before December 2021). Then, each of the publications was carefully reviewed. After we filtered the irrelevant papers, the ncFO collected 90 entries of ncRNA–ferroptosis associations, which included 17 circRNAs, 46 miRNAs, and 21 lncRNAs across different species. The detailed information of each entry contains the ncRNA name, ncRNA identifier (ID), species, disease, tissue/cell line, regulation, target, experiment, and PubMed identifier (PMID) (Figure 1).

### Prediction of ferroptosis-associated ncRNAs

To extend the potential ncRNAs involved in ferroptosis, we proposed “Prediction tools” to predict ncRNA–ferroptosis associations. In the current ncFO database, we predicted the potential ncRNAs associated with ferroptosis through regulation and co-expression between ncRNAs and known ferroptosis genes (Figure 1). We first obtained 259 known ferroptosis genes from FerrDb [10], including 108 ferroptosis driver genes, 69 ferroptosis suppressor genes, and 111 ferroptosis marker genes, some of which play diverse roles in the ferroptosis process. Next, the potential ferroptosis-associated ncRNAs were predicted through the mutual regulation between ncRNAs and known ferroptosis genes. The ncRNA–gene regulations were extracted from the curated databases, including miRTarBase [11], TarBase [12], miRecords [13], TransmiR [14], LncRNA2Target [15], LncTarD [16], and LncReg [17]. It is worth noting that the miRNAs involved in transcription factor (TF) regulation of miRNAs are precursor miRNAs, while the miRNAs involved in miRNA regulation of genes are mature miRNAs. We retained the ncRNAs that regulate ferroptosis genes. The regulatory networks were visualized using *Cytoscape.js* [18]. As a result, we obtained 3260 predicted entries, including 598 miRNAs and 178 lncRNAs. Furthermore, we predicted the potential ferroptosis-associated ncRNAs through their co-expression with known ferroptosis genes in specific cancer types. The gene and miRNA expression profiles were downloaded from UCSC xena (https://xenabrowser.net/datapages/). The lncRNA expression profiles were downloaded from TANRIC (https://www.tanric.org/). Likewise, the miRNAs/lncRNAs co-expressed with ferroptosis genes in a specific cancer type were preserved. At the default threshold of correlation coefficient 0.3 and *P* value 0.05, we obtained 1,104,319 and 1,488,342 predicted entries, including 967 miRNAs and 9632 lncRNAs, by Pearson and Spearman correlation coefficients, respectively, in more than 8000 samples across 20 cancer types. On average, there were 9.55 miRNAs and 220.01 lncRNAs co-expressed with one ferroptosis gene in the Pearson correlation results. Users could also define the parameters as needed to filter the query results. In addition, the ncFO database enables users to perform online survival analysis and differential gene expression analysis of ncRNAs by implementing the R language.

## Database content and user interface

**Figure 2:**
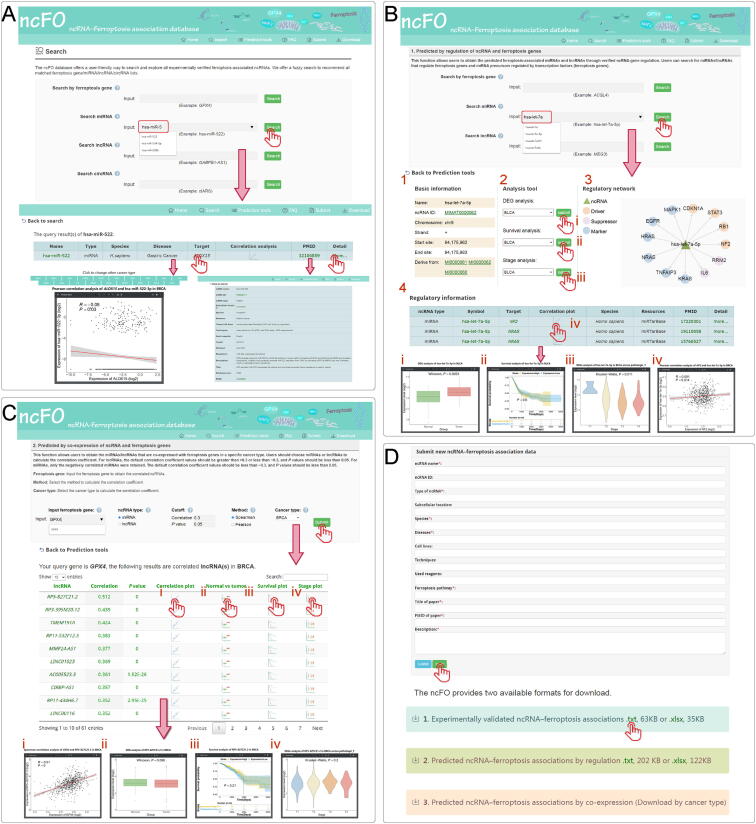
**A comprehensive view of ncFO database** **A.** Interface of the “Search” page. Users could search by miRNA, lncRNA, or circRNA. Here, using hsa-miR-522 as an example, we obtained experimentally verified entries. **B.** Interface of the “Predicted by regulation” function. Here, using hsa-let-7a-5p as an example, we obtained ncRNA–ferroptosis gene regulation information and performed an online analysis of the queried ncRNAs. **C.** Interface of the “Predicted by co-expression” function. Here, using *GPX4* as an example, we calculated the ncRNAs correlated with *GPX4* in a specified cancer type. In addition, we performed an online analysis of the correlated ncRNAs. **D.** Interfaces of the “Submit” and “Download” pages. Users can submit novel ferroptosis-associated ncRNAs to ncFO. In addition, all of the experimentally verified entries and predicted entries could be downloaded freely. DEG, differentially expressed gene.

The ncFO database offers a user-friendly interface for searching, predicting, and downloading all ferroptosis-associated ncRNAs freely. (1) On the “Home” page, users can glance over the brief introduction about the ncFO database and the statistics of the ncRNAs. (2) On the “Search” page, users can search all the experimentally verified entries by ferroptosis genes and three kinds of ncRNAs (miRNA, lncRNA, and circRNA). We offer a fuzzy search to recommend all matched ferroptosis gene/miRNA/lncRNA/circRNA lists (Figure 2A). In the returned results, we offer a convenient way for users to perform an online correlation analysis of ncRNA and target genes with expression profiles in The Cancer Genome Atlas (TCGA) dataset. In addition, users can click “more” to display the detailed information of the corresponding entry. (3) On the “Prediction tools” page, users can predict ferroptosis-associated ncRNAs using two different methods. First, users can search by ferroptosis genes, miRNAs, or lncRNAs to obtain miRNAs/lncRNAs that regulate ferroptosis genes. Similarly, we also offer a fuzzy search to return all matched ferroptosis gene/miRNA/lncRNA lists (Figure 2B). The search results contain basic information, analysis tools, regulatory networks, and detailed information on regulations. Additionally, we could perform online correlation analysis for the query ncRNA and the target genes in a specified cancer type based on the TCGA dataset. Second, we permitted users to predict ferroptosis-associated ncRNAs using co-expression analysis (Figure 2C). Users need to input the ferroptosis genes, ncRNA type, cutoff, method, and specify the cancer type. Then, ncFO returns the co-expressed ncRNAs based on the input parameters. We implemented R language for online analysis of the ncRNAs in the TCGA dataset, including gene expression boxplots, survival plots, and correlation plots. (4) On the “Download” page, users can download all of the experimentally verified and predicted ncRNAs associated with ferroptosis (Figure 2D). Additionally, the website provides two available formats for download (Text and Excel). Furthermore, ncFO strongly encourages users to submit novel experimentally supported ncRNA–ferroptosis associations.

## Discussion

In conclusion, the ncFO database has peculiar innovation in the following aspects: (1) providing the manually curated ncRNA–ferroptosis associations in cancer, (2) proposing “Prediction tools” based on ncRNA regulation and co-expression analysis in a specific cancer type, and (3) offering an online web server for convenient searching, querying, and analyzing of ferroptosis-associated ncRNAs. In the future, we will continue to collect scientific literature on ferroptosis-associated ncRNAs and regularly update the database. Moreover, we will improve our database by adding other practical tools, such as a collection of small molecules that target ferroptosis-associated ncRNAs. We sincerely hope that the ncFO database will provide a reliable and convenient platform for researchers to investigate ferroptosis, which will be helpful for identifying drug targets and ultimately benefit cancer therapy.

## Data availability

All data obtained and/or analyzed in this study are available in ncFO at http://www.jianglab.cn/ncFO/.

## Competing interests

The authors have declared no competing interests.

## CRediT authorship contribution statement

**Shunheng Zhou:** Conceptualization, Visualization, Methodology, Writing – original draft. **Yu’e Huang:** Visualization, Methodology, Writing – original draft. **Jiani Xing:** Data curation. **Xu Zhou:** Software. **Sina Chen:** Investigation, Validation. **Jiahao Chen:** Investigation, Validation. **Lihong Wang:** Conceptualization, Project administration, Writing – review & editing, Supervision. **Wei Jiang:** Conceptualization, Project administration, Writing – review & editing, Supervision. All authors have read and approved the final manuscript.
